# CPI-637 as a Potential Bifunctional Latency-Reversing Agent That Targets Both the BRD4 and TIP60 Proteins

**DOI:** 10.3389/fcimb.2021.686035

**Published:** 2021-07-19

**Authors:** Tengyi Zheng, Pei Chen, Yifan Huang, Jiayin Qiu, Chenliang Zhou, Ziyao Wu, Lin Li

**Affiliations:** ^1^ Guangdong Provincial Key Laboratory of New Drug Screening, Guangzhou Key Laboratory of Drug Research for Emerging Virus Prevention and Treatment, School of Pharmaceutical Sciences, Southern Medical University, Guangzhou, China; ^2^ School of Pharmaceutical Science, Zhejiang Chinese Medical University, Hangzhou, China

**Keywords:** CPI-637, HIV-1 functional cure, BRD4 inhibitor, TIP60 inhibitor, HIV-1 latency-reversing agent

## Abstract

The failure of highly active antiretroviral therapy (HAART) has been largely responsible for the existence of latent human immunodeficiency virus type 1 (HIV-1) reservoirs. The “shock and kill” strategy was confirmed to reactivate HIV-1 latent reservoirs by latency-reversing agents (LRAs) for accelerated HIV-1 clearance. However, a single LRA might be insufficient to induce HIV-1 reactivation from latency due to the complexity of the multiple signaling regulatory pathways that establish the HIV-1 latent reservoir. Therefore, combinations of LRAs or dual-mechanism LRAs are urgently needed to purge the latent reservoirs. We demonstrate here for the first time that a dual-target inhibitor with a specific suppressive effect on both BRD4 and TIP60, CPI-637, could reactivate latent HIV-1 *in vitro* by permitting Tat to bind positive transcription elongation factor b (P-TEFb) and assembling Tat-super-elongation complex (SEC) formation. In addition, CPI-637-mediated TIP60 downregulation further stimulated BRD4 dissociation from the HIV-1 long terminal repeat (LTR) promoter, allowing Tat to more effectively bind P-TEFb compared to BRD4 inhibition alone. Much more importantly, CPI-637 exerted a potent synergistic effect but alleviated global T cell activation and blocked viral spread to uninfected bystander CD4^+^ T cells with minimal cytotoxicity. Our results indicate that CPI-637 opens up the prospect of novel dual-target inhibitors for antagonizing HIV-1 latency and deserves further investigation for development as a promising LRA with a “shock and kill” strategy.

## Introduction

Although highly active antiretroviral therapy (HAART) has dramatically decreased morbidity and mortality in patients infected with human immunodeficiency virus type 1 (HIV-1), a rapid rebound in plasma viremia to pretreatment levels is observed after the interruption of HAART treatment (Van Lint et al., 2013). Latent HIV-1 cellular reservoirs may contribute to the persistence of HIV-1 and promote the emergence of resistant mutants (Wallet et al., 2019; Ventura, 2020). Due to the lack of viral transcription, latently infected cells are usually difficult to identify and eliminate by both the innate and adaptive immune responses and antiviral drugs (Chun et al., 1997; Dufour et al., 2020). One strategy, termed “shock and kill”, aims to reactivate dormant viruses from the HIV-1 latent reservoir by regulating host-dependent pathways and then eradicate the reservoir through HIV-1-related cytopathic effects or immune system-based clearance of infected cells (Abner and Jordan, 2019).

To expose the latent reservoirs for accelerated clearance, numerous latency-reversing agents (LRAs) targeting specific steps of HIV-1 transcription have been identified (Spivak and Planelles, 2018). However, commonly employed LRAs have been suggested to be toxic, mutagenic or ineffective in clinical trials involving large sample sizes and prolonged treatment. Moreover, some LRAs might not reactivate the total HIV-1 reservoir due to the broad integrational landscape of the provirus, condensed chromatin state of resting T cells, sequestration of necessary transcription factors and physiological heterogeneity of the host cells (Spivak and Planelles, 2018; Zhao et al., 2019). Therefore, a combinatory regimen including a set of LRAs targeting multiple steps of HIV-1 latency is expected to reactivate all integrated proviruses and effectively purge latent reservoirs.

There are many viral and cellular proteins involved in HIV-1 transcription that may represent potential targets of LRAs. One of the best studied proteins is BRD4, a member of the bromodomain (BRD) and extraterminal domain (BET) protein family (Banerjee et al., 2012; Zhu et al., 2012; Li et al., 2013; Lu et al., 2016). The binding of BRD4 to chromatin may regulate HIV-1 gene expression, especially transcription elongation from HIV-1 promoter long terminal repeats (LTRs). Positive transcription elongation factor b (P-TEFb), an activation-dependent transcription factor and essential cofactor for HIV-1 Tat, is composed of cyclin-dependent kinase 9 (CDK9) and cyclin T1. BRD4 competes with Tat for P-TEFb binding and inhibits the activation of transcription elongation. Therefore, BRD4 is a key regulator of the establishment of HIV-1 latency, and BET inhibitors provide an effective way to reactivate latent HIV-1 by favoring the recruitment of P-TEFb by Tat to the HIV-1 promoter LTR (Itzen et al., 2014).

Histone acetyltransferases (HATs) and histone deacetylases (HDACs) are enzymes responsible for the modification of specific lysine residues in core histones and thought to be key modifiers of chromatin structures. HIV-1-Tat interactive protein (TIP60), also termed KAT5, is one of the major HATs known to modify H4 and plays an essential role in transcriptional regulation (Kimura and Horikoshi, 1998). Acetyl-histone H3 (AcH3) and acetyl-histone H4 (AcH4) interact with BRD4 and regulate its recruitment to the HIV-1 LTR. A recent study showed that downregulating the expression or activity of TIP60 removed the acetyl group from AcH4 and BRD4 from the HIV-1 LTR, resulting in enhanced super-elongation complex (SEC) loading, Tat transactivation and HIV-1 reactivation. The combination of MG-149 (a TIP60 inhibitor) and JQ1 (a BRD4 inhibitor) promoted HIV-1 reactivation in activated cell lines and quiescent primary T cells from HIV-1-infected patients treated with HAART (Li et al., 2018). Therefore, the TIP60-AcH4-BRD4 axis might be an effective new target for reactivating the HIV-1 latent reservoir.

A highly homologous pair of BRD-containing transcriptional coactivators, HATs cyclic-AMP response element binding protein (CBP) and adenoviral E1A-binding protein of 300 kDa (EP300), were reported to be associated with multiple disease pathways (Dutta et al., 2016). The small-molecule compound CPI-637 is a novel and selective inhibitor that targets CBP/EP300 and BRD4 (Taylor et al., 2016). In the present study, we were delighted to find that CPI-637 effectively reactivated latent HIV-1 *in vitro* by dissociating BDR4 from the HIV-1 promoter, recruiting Tat to stimulate HIV-1 elongation. Moreover, CPI-637-mediated TIP60 downregulation further stimulate BRD4 dissociation from the HIV-1 LTR promoter *via* AcH3 upregulation and AcH4 downregulation, allowing Tat to more effectively bind P-TEFb and facilitating Tat-SEC formation compared to BRD4 inhibition alone. CPI-637 might be an ideal and bifunctional candidate LRA that targets both BRD4 and TIP60 with a shock-and-kill strategy for a HIV-1 functional cure.

## Materials and Methods

### Cell Culture

J-Lat A2 cells (Jurkat T cells containing an HIV-1 5′-LTR-Tat-Flag-iRES(internal ribosome entry site)-EGFP-3′-LTR construct), J-Lat 10.6 cells (Jurkat T cells harboring a full-length integrated HIV-1 genome and expressing green fluorescent protein (GFP) upon activation) and ACH2 cells (A3.01 T cells chronically infected with HIV-1 with transactivation response (TAR) mutation) were kindly provided by Dr. Shibo Jiang and Dr. Lu Lu of Fudan University (Shanghai, China). Those latently infected cell lines were grown at 37°C with 5% CO_2_ in RPMI 1640 medium (Gibco, Grand Island, NY, USA) with 10% fetal bovine serum (FBS, Gibco, USA) and 1% penicillin/streptomycin (Invitrogen, Carlsbad, CA, USA). TZM-bl cells were purchased from ATCC (Manassas, VA) and cultured at 37°C with 5% CO_2_ in Dulbecco’s modified Eagle’s medium (DMEM) with 10% FBS and 1% penicillin/streptomycin.

### Materials

An antibody specific for HIV-1 p24 (183-H12-5C, mouse, 1:2000) was obtained from the National Institutes of Health AIDS Research and Reference Reagent Program. Antibodies specific for BRD4 (13440, rabbit, 1:1000), CDK9 (2316, rabbit, 1:1000), cyclin T1 (81464, rabbit, 1:1000), the Rpb1 C-terminal domain (CTD, 2629, rabbit, 1:1000), p-Rpb1 CTD (Ser2, 13499, rabbit, 1:1000), AcH3K9 (9649, rabbit, 1:1000), AcH4K8 (2594, rabbit, 1:1000), AcH3K14 (7627, rabbit, 1:1000) and β-actin (3700, mouse, 1:1000) were purchased from Cell Signaling Technology (CST, USA). Antibody specific for Tat (ab6539, rabbit, 1:3000) was purchased from Abcam (UK). Antibodies specific for p-CDK9 (Thr186, sc-139604, rabbit, 1:1000), TIP60(sc-166323, rabbit, 1:1000) and AcH2AK5 (GT1262, mouse, 1:500) were purchased from Santa Cruz Biotechnology (USA) and GeneTex (USA), respectively. Human anti-CD25-FITC (555431, mouse, 1:100), anti-CD69-FITC (557049, mouse, 1:100), anti-HLA-DR-FITC (556643, mouse, 1:100), and anti-CD38-FITC (555459, mouse, 1:100) antibodies and anti-CD4-FITC (561842, mouse, 1:100), anti-CCR5-APC (550856, mouse, 1:100) and anti-CXCR4-APC (560936, mouse, 1:100) antibodies were obtained from BD Biosciences (USA). CPI-637, JQ1, SAHA and MG-149 were purchased from MedChemExpress (MCE, Finland), and prostratin was purchased from Sigma (USA). All reagents were diluted in dimethyl sulfoxide (DMSO, Sigma, USA).

### Flow Cytometry

GFP fluorescence was measured in a BD FACSCanto II flow cytometer (USA). The data were analyzed *via* FlowJo software (TreeStar, USA). J-Lat A2 and J-Lat 10.6 cells (5×10^5^ cells/well) were incubated with CPI-637, MG-149, JQ1, SAHA or prostratin at the indicated concentrations for 48 h or the indicated durations in 48-well plates, and the percentage of GFP^+^ cells was used as a standard to indicate HIV-1 reactivation.

### Enzyme-Linked Immunosorbent Assay (ELISA)

ACH2 cells (5×10^5^ cells/well) were seeded into 96-well plates and incubated with CPI-637, MG-149 or JQ1 at different concentrations. Then, the HIV-1 p24 antigen levels in the ACH2 cell supernatants were analyzed by ELISA.

### RNA Extraction, Reverse Transcription, and Real-Time Quantitative PCR (RT-qPCR)

Total RNA was extracted using TRIzol (Invitrogen, USA), and cDNA was amplified from 50 to 100 ng of RNA using the Prime Script RT Reagent Kit (TaKaRa, Japan). Gene products were analyzed by qPCR using SYBR Select Master Mix (TaKaRa) in a LightCycler 480 machine (Roche, Switzerland) in 96-well plates. The effects of CPI-637, MG-149, SAHA, prostratin and JQ1 treatment for 48 h on HIV-1 gene expression in ACH2 cells were assayed by RT-qPCR. The primers used for qPCR amplification are listed in **Table 1**.

**Table 1:** RT-PCR primer sequences.

Sequences		
GAPDH	Forward	CTCTGCTCCTCCTGTTCGAC
	Reverse	AGTTAAAAGCAGCCCTGGTGA
Gag	Forward	GTCCAGAATGCGAACCCAGA
	Reverse	GTTACGTGCTGGCTCATTGC
Tat	Forward	ATGGAGCCAGTAGATCCTAGACT
	Reverse	CGCTTCTTCCTGCCATAGGA
Vpr	Forward	CCACAAAGGGAGCCATACAATG
	Reverse	TTATGGCTTCCACTCCTGCC
Vif	Forward	CACACAAGTAGACCCTGACCT
	Reverse	CCCTACCTTGTTATGTCCTGCT
LTR	Forward	GCCTCCTAGCATTTCGTCACAT
	Reverse	GCTGCTTATATGTAGCATCTGAGG
IL-1β	Forward	CAACAGGCTGCTCTGGGATT
	Reverse	GGGCCATCAGCTTCAAAGAAC
IL-6	Forward	GTAGCCGCCCCACACAGA
	Reverse	CATGTCTCCTTTCTCAGGGCTG
TNF-α	Forward	CCCAGGGACCTCTCTCTAATCA
	Reverse	GCTTGAGGGTTTGCTACAACATG
MCP-1	Forward	CAGCCAGATGCAATCAATGCC
	Reverse	TGGAATCCTGAACCCACTTCT
TIP60	Forward	AACCAGGACAACGAAGATGAG
	Reverse	GTCACCCATTCATCCAGACG

### Cytotoxicity Assay

Peripheral blood mononuclear cells (PBMCs) from healthy individuals, J-Lat cells, TZM-bl and ACH2 cells were placed in 96-well plates (5×10^5^ cells/well) and treated with CPI-637 for the cytotoxicity assay. After incubation for 48 h at 37°C, cell viability was measured using an MTT (3–2,5 diphenyl tetrazolium bromide, Sigma, USA) assay. The colored formazan products were solubilized with DMSO and photometrically measured at 570 nm in a multiwell plate reader (Bio-Rad Laboratories, USA). The 50% cytotoxic concentration (CC_50_) was calculated with CalcuSyn software.

### Detection of T Cell Activation Markers and HIV-1 Receptors/Coreceptors

PBMCs (1×10^6^ cells/well) isolated from the blood of healthy donors at Nanfang Hospital by standard density gradient centrifugation with Histopaque 1077 (Sigma, USA) were seeded in 6-well plates. After incubation with CPI-637 for 24 h, 48 h or 72 h, the PBMCs were stained with anti-CD25-FITC, anti-CD69-FITC, anti-HLA-DR-FITC, anti-CD38-FITC, anti-CD4/anti-CCR5 or anti-CD4/anti-CXCR4 antibodies (BD Bioscience, USA) at 4°C for 30 min in the dark. Then, the effects of CPI-637 on T cell activation were analyzed with a BD FACSCanto II flow cytometer.

### Western Blot (WB) Analysis

ACH2 cells (1×10^6^ cells/well) were treated with CPI-637 and JQ1 at different concentrations or for various durations. Approximately 50–150 mg of thermally denatured cellular lysates was used for SDS-polyacrylamide gel electrophoresis (SDS-PAGE) immunoblot analysis. The PVDF membrane (Roche, USA) was incubated with antibody. Subsequently, the immunoblot bands with enhanced chemiluminescence (ECL) substrates (CST, USA) were exposed to film for image development.

### Anti-Inflammatory Effects of CPI-637

THP-1 cells (5 × 10^5^ cells/well) were treated with 10 ng·ml^−1^ phorbol 12-myristate 13-acetate (PMA, Sigma, USA) for 24 h in 6-well plates. After incubation with CPI-637 for 24 h, the THP-1 cells were collected for RNA extraction and the measurement of proinflammatory cytokine expression by RT-qPCR as described above.

### Coimmunoprecipitation (co-IP)

ACH2 cells (1×10^6^ cells/well) were treated with CPI-637 or JQ1 for 48 h. Then, precleared protein extracts of ACH2 cells were added to protein A/G agarose with anti-BRD4 antibody (CST) and incubated on a rotator at 4°C overnight. The beads were washed with cold IP lysis buffer containing 0.1% phenylmethylsulfonyl fluoride (PMSF), eluted with 0.1 M glycine and analyzed by WB analysis with the indicated antibodies.

### Chromatin Immunoprecipitation (ChIP)

ChIP assays were performed with kits (CST, USA) according to the manufacturer’s protocol and previously described procedures (Liang et al., 2019). Briefly, ACH2 cells (1 × 10^6^ cells/well) were fixed in 1% formaldehyde. Chromatin was sonicated into fragments 200–500 nucleotides in length and subjected to immunoprecipitation. DNA fragments were incubated with antibodies specific for IgG, CDK9, BRD4, TIP60, Tat, or Pol II CTD-Ser2P at 4°C overnight. After incubation with 50 µl of protein G agarose beads, the immunocomplexes were washed, the chromatin was eluted and reverse cross-linked at 65°C overnight. The DNA was extracted, and qPCR was performed using SYBR Green Mix on a LightCycler 480 machine (Roche, Switzerland). The upstream primer sequence was 5′-AGCTTGCTACAAGGGACTTTCC-3′, and the downstream primer sequence was 5′-GTGGGTTCCCTAGTTAGCCAGAG-3′. The amounts of the fragments after incubation with the antibodies above were normalized against the input DNA and are presented as % the input DNA.

### Luciferase Assays

TZM-bl cells (5×10^5^ cells/well) were seeded into a 48-well plate. PNL4-3-luc and pcDNA 3.1(+)-Tat were mixed with Lipofectamine 3000, followed by coincubation of this mixture with the cells for 48 h. The cells were lysed, and relative luciferase activity was detected using a Dual-Luciferase Reporter Assay System (Promega) according to the manufacturer’s instructions. Substrate luminescence was determined by scanning using a microplate reader with a white plate. Relative transcription activity was normalized against Renilla luciferase activity and then compared with relative transcription activity in the PNL4-3-luc group (Zhang et al., 2019).

### Synergistic Reactivation of Latent HIV-1 Expression by CPI-637 and LRAs

We used the Bliss independence model as a metric to evaluate the latency-reversing activity of combinations of drugs (Goldoni and Johansson, 2007). Suppose for two drugs, x and y, that *f*_axy, P_ is the predicted fraction affected by a combination of drugs x and y according to the experimentally observed fraction individually influenced by drug x (ƒ_ax_) and drug y (ƒ_ay_). This value is defined by the equation ƒ_axy, P_ = ƒ_ax_ + ƒ_ay_ − (ƒ_ax_)(ƒ_ay_). Based on the Bliss model, ƒ_axy, O_, the observed combined fraction, was compared with ƒ_axy, P_ with the equation (Δƒ_axy_ = ƒ_axy, O_ − ƒ_axy, P_). When Δ*f*_axy_ > 0, the combination of LRAs displayed synergy. When Δ*f*_axy_ < 0, then the combined effects of the two LRAs showed antagonism.

### Statistical Analysis

All the experimental results are presented as the mean ± SD of at least three independent experiments, and all statistical analyses were calculated with software GraphPad Prism 6.0 (San Diego, CA, USA). Moreover, one-way analysis of variance (ANOVA) followed by Dennett’s multiple comparison *post hoc* test was used to assess differences between the groups. P-values below 0.05 were considered to demonstrate statistically significant differences. Statistical significance was defined as *p < 0.05 and highly statistical significance as **p < 0.01.

## Results

### CPI-637 Induces HIV-1 Expression in Latently Infected Cell Lines *In Vitro*

**Figure 1:**
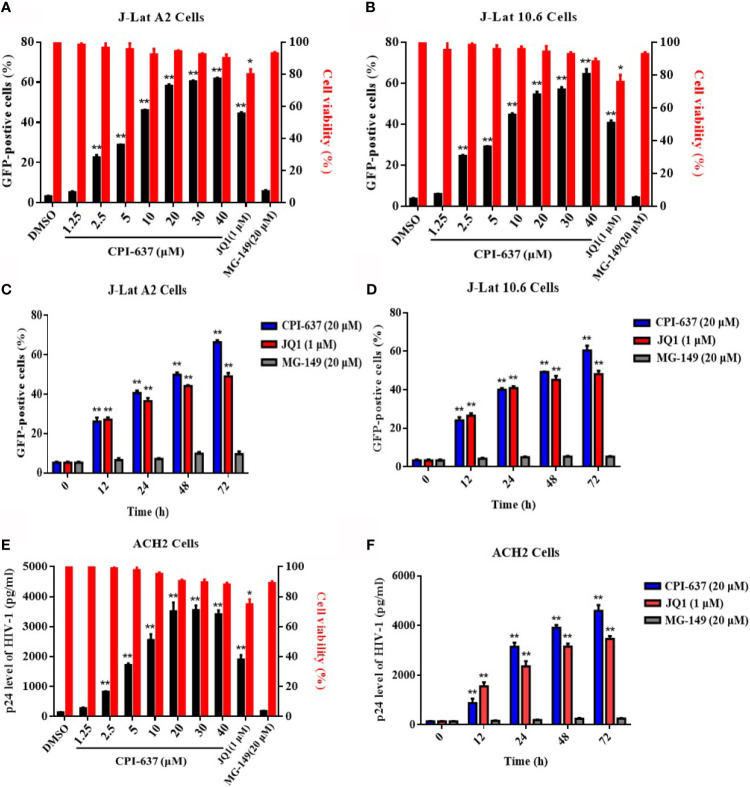
CPI-637 induces HIV-1 expression in latently infected cell lines in *vitro*. J-Lat A2 and J-Lat 10.6 cells were treated with CPI-637 for 48 h at the indicated concentrations **(A, B)** or with CPI-637 (20 μM) at the indicated time points **(C, D)**. And the percentage of GFP-positive cells within the entire population, representing the level of HIV-1 transcription **(A–D)**, was measured by flow cytometry and the cell viability **(A**, **B)** was detected by MTT assay, respectively. ACH2 cells were treated with CPI-637 at the indicated concentrations **(E)** or the indicated time points **(F)**. HIV-1 p24 antigen expression **(E**, **F)** was detected by ELISA and the cell viability **(E)** was detected by MTT assay, respectively. (*p < 0.05 vs DMSO control group, **p < 0.01 vs DMSO control group).

Two well-established latently infected Jurkat T cell lines (J-Lat A2 and J-Lat 10.6 cells) were chosen to investigate the potential effect of CPI-637 on the reactivation of latent HIV-1 expression; these cell lines contain a latent and transcriptionally competent HIV-1 provirus with a GFP reporter gene. The percentage of GFP-positive cells (GFP^+^ %) within the entire population represented latent HIV-1 activation and was determined by flow cytometry. As shown in **Figures 1A–D**, CPI-637 significantly increased GFP^+^ % in a dose- and time-dependent manner in both J-Lat A2 and J-Lat 10.6 cells. Furthermore, the reactivation by CPI-637 was confirmed in ACH2 cells chronically infected with HIV-1. The production of HIV-1 p24 antigen in the supernatants of ACH2 cells in the presence or absence of CPI-637 was detected by ELISA. Correspondingly, CPI-637 clearly promoted HIV-1 p24 expression with prolonged treatment time and increasing concentrations of CPI-637 (**Figures 1E, F**). In the experiment, JQ1 (a BRD4 inhibitor) and MG-149 (a TIP60 inhibitor) were used as controls. Consistent with reported studies, JQ1 at 1 μM reversed HIV-1 latency in all tested latent HIV-1-infected cells, but MG-149 had no effect on HIV-1 latency.

**Figure 2:**
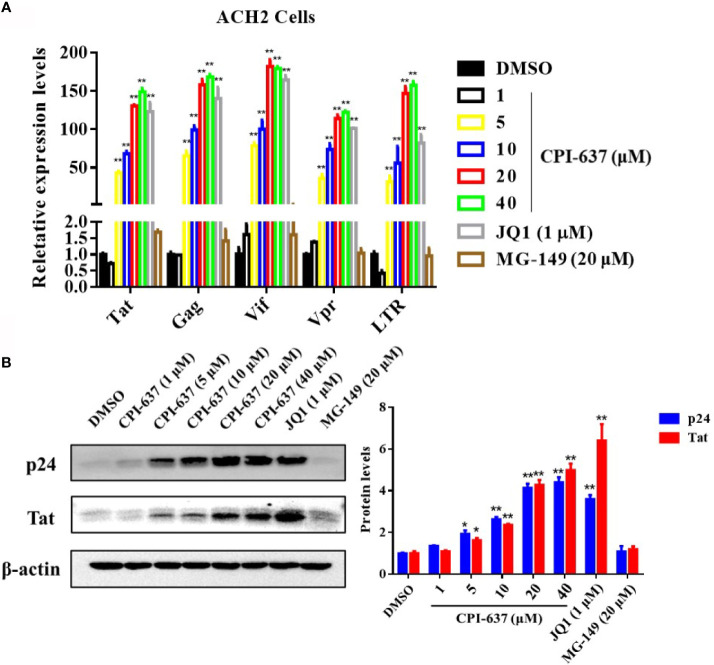
CPI-637 increases the HIV-1 transcriptional gene and core protein expression in *vitro*. ACH2 cells were treated with CPI-637 at the indicated concentrations for 48 h. HIV-1 RNA levels were analyzed by RT-qPCR **(A)**. The relative expression levels were compared to those in DMSO control group. The expression levels of Tat and p24 proteins were analyzed by WB **(B)**, and the quantifications of Tat and p24 were also performed in **(B)** (*p < 0.05 vs DMSO control group, **p < 0.01 vs DMSO control group).

The effects of CPI-637 on the transcriptional expression of HIV-1 genes (Tat, Gag, Vif, Vpr and R and U5 regions at the HIV-1 5’ LTR) were also determined by RT-qPCR. Notably, CPI-637 efficiently increased the transcriptional expression of all tested HIV-1 genes in full-length HIV-1 transcripts in a dose-dependent manner (**Figure 2A**). We further investigated the expression of HIV-1 core nucleocapsid protein p24 and a key transactivator Tat protein on ACH-2 cells in the presence or absence of CPI-637 by WB assay. As shown in **Figure 2B**, the expression of HIV-1 p24 and Tat proteins were also increased by CPI-637 in a dose-dependent manner. These results further confirmed that CPI-637 reverses latent HIV-1 infection in latently infected cells.

### CPI-637 Has Low Toxicity *In Vitro*

The failure of some LRAs in clinical trials indicates that low toxicity is necessary for an ideal candidate LRA. Here, we detected the cytotoxicity of CPI-637 in both latently infected HIV-1 cell lines (J-Lat A2, J-Lat 10.6 and ACH2) and HIV-1 target cells (TZM-bl and human PBMCs isolated from healthy donors) by MTT assay. As shown in **Table 2**, compared with JQ1 and MG-149, CPI-637 displayed decreased cytotoxicity in all tested cell lines, with CC_50_ values ranging from 195.12 to 303.60 μM. The CC_50_ values of CPI-637 were approximately 10 times greater than those of JQ1, suggesting that CPI-637 has strong development potential as an ideal candidate LRA.

**Table 2:** *In vitro* cytotoxicity of CPI-637.

Cells		CC_50_ (μM)	
	CPI-637	JQ1	MG-149
hPBMC	291.60 ± 2.04	27.44 ± 1.65	204.57 ± 3.61
J-Lat A2	195.12 ± 1.11	26.93 ± 1.35	96.41 ± 4.13
J-Lat 10.6	203.47 ± 0.98	23.26 ± 1.43	107.30 ± 3.51
ACH2	225.51 ± 1.27	24.33 ± 1.45	127.72 ± 1.96
TZM-bl	303.60 ± 2.17	34.19 ± 2.04	191.95 ± 4.47

Data were presented in means ± SD.

**Figure 3:**
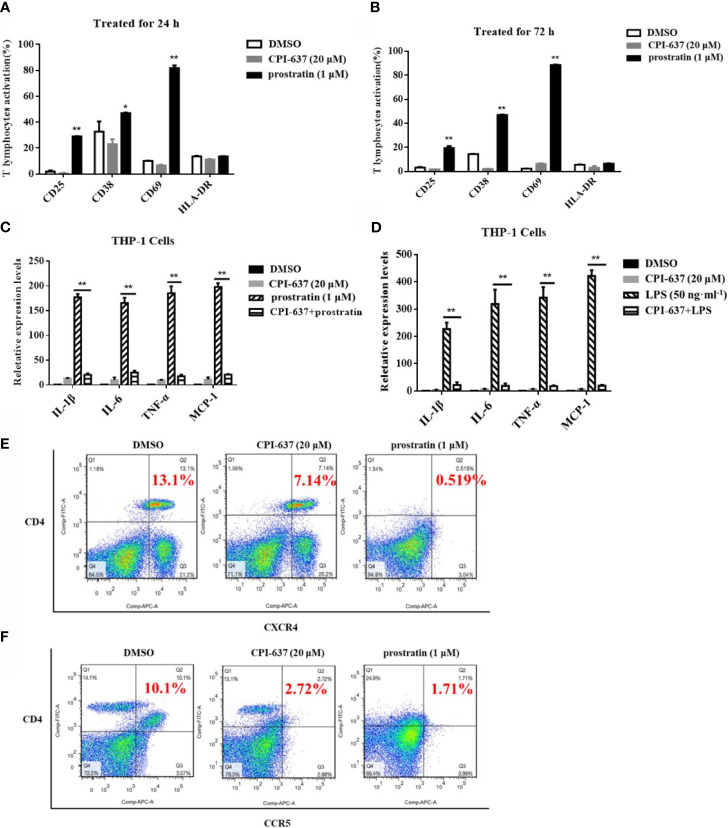
CPI-637 does not up-regulate HIV-1 receptor/coreceptors and does not induce global T-cell activation. After treatment with CPI-637 (20 μM) for 24 **(A)** or 72 h **(B)**, the expressions of CD69, CD38, CD25 and HLA-DR in PBMCs from healthy HIV-negative donors were analyzed by flow cytometry. (*p < 0.05 vs DMSO control group, **p < 0.01 vs DMSO control group) After treatment with CPI-637 (20 μM) for 48 h, the expression of pro-inflammatory cytokines, including IL-1β, IL-6, TNF-α and MCP-1, in THP-1 cells were analyzed by RT-qPCR **(C**, **D)**. And fractions of CD4, CXCR4 **(E)** and CCR5 **(F)** positive cells in PBMCs were analyzed by flow cytometry. (*p < 0.05 vs prostratin group, **p < 0.01 vs prostratin group).

The propensity to nonspecifically activate bystander T cells has been suggested to obstruct the applied development of current LRAs in patients due to the potential induction of proinflammatory cytokine release (Bullen et al., 2014). Accordingly, we first examined the effects of CPI-637 on global T cell activation among human PBMCs by flow cytometry. As shown in **Figures 3A, B**, CPI-637 caused different T cell markers (CD69, CD38, CD25 and HLA-DR) to be expressed at levels similar to those observed in human PBMCs treated with DMSO control for both 24 h and 72 h. Furthermore, release of the proinflammatory cytokines IL-1β, IL-6, TNF-α and MCP-1 in the presence and absence of CPI-637 was determined by RT-qPCR. Our results showed that CPI-637 at 20 μM did not affect the relative expression of proinflammatory cytokines in THP-1 cells (**Figures 3C, D**). Consistent with the literature (Hashemi et al., 2018), a protein kinase C (PKC) agonist, prostratin, at 1 μM significantly increased the levels of T cell activation biomarkers and upregulated the release of proinflammatory cytokines following stimulation for 24 or 72 h, indicating that it may produce serious toxicity by affecting general T cell activation in clinical trials (**Figures 3A–D**). Notably, CPI-637 significantly decreased the release of inflammatory cytokines induced by prostratin in THP-1 cells, indicating that CPI-637 may reduce the side effects of PKC agonists when combined with LRAs (**Figure 3C**).

### CPI-637 Downregulates HIV-1 Receptors/Coreceptors

Generally, HIV-1 virions enter to the host cells by binding to the cell-surface CD4 receptor and CCR5/CXCR4 co-receptors. These receptor/co-receptors are expressed at the cell surface of CD4^+^ T lymphocytes, which serve as the primary targets of HIV-1, but are also present on dendritic cells and macrophages. Some LRAs downregulate the expression of HIV-1 receptors/coreceptors, which may avoid the risk of increased susceptibility to HIV-1 infection during the reactivation of HIV-1 latency (Benamar et al., 2009; Grande et al., 2019). Human PBMCs, containing lymphocytes, monocytes and dendritic cells, were used for HIV-1 infected primary target cells. Our results also confirmed that CPI-637 downregulated the expression of a CD4 receptor/coreceptor (CCR5 and CXCR4) on the surface of PBMCs, similar to prostratin (**Figures 3E, F**). We further detected the infectious activities in isolated hPBMCs by reactivated latently HIV-1 on ACH-2 cells treated with CPI-637 for 48 h. CPI-637 could not obviously decrease the HIV-1 p24 expression in PBMCs, indicating that it might not clearly reduce the risk of reactivated HIV-1 infection in bystander cells even though it downregulated the expression of a CD4 receptor/coreceptor (CCR5 and CXCR4) on the surface of PBMCs (Data not shown).

### CPI-637 Promotes Latent HIV-1 Expression *via* a Tat-Dependent P-TEFb Pathway

**Figure 4:**
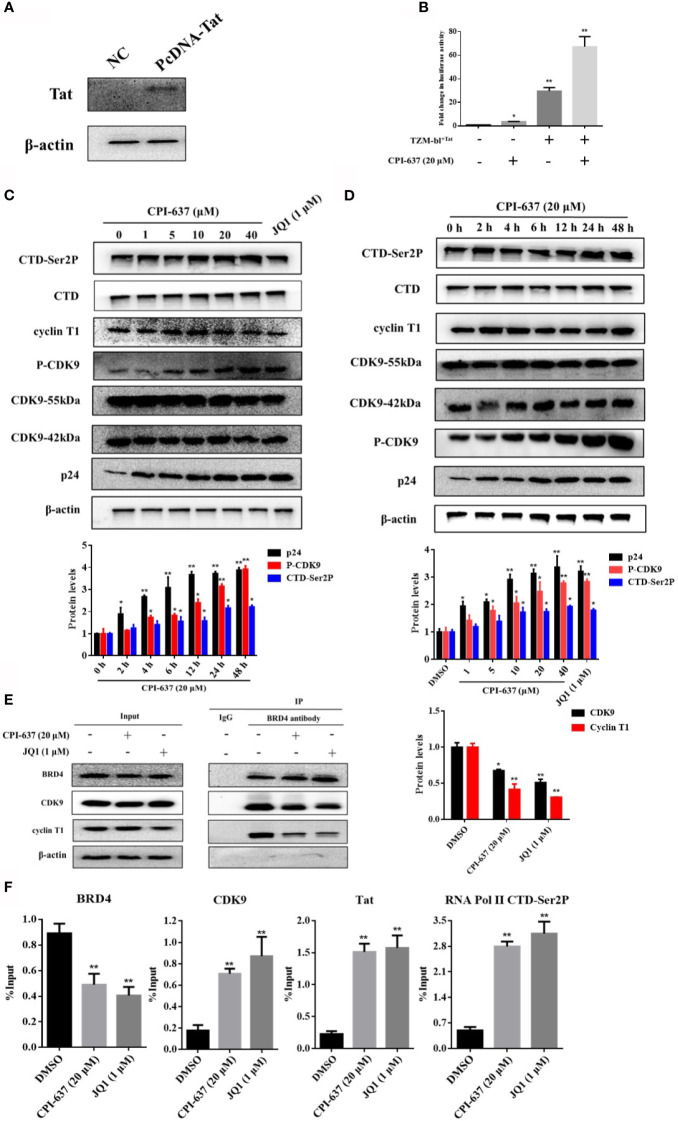
CPI-637 promotes latent HIV-1 expression *via a* Tat-dependent P-TEFb pathway. TZM-bl cells containing an integrated HIV LTR-luciferase construct were nucleofected with a plasmid expressing Tat (+) or a negative control plasmid (−). And the expression of TIP60 in TZM-bl^+Tat^ cells was detected by WB **(A)**. TZM-bl^+Tat^ Cells were treated with CPI-637 (20 μM) for 48 h and subjected to luciferase assay **(B)**. ACH2 cells were treated with CPI-637 for 48 h at the indicated concentrations **(C)** or with CPI-637 (20 μM) at the indicated time points **(D)**, and then the expressions of CTD, CTD-Ser 2P, cyclin T1, CDK9, p-CDK9 and p24 were analyzed by WB. ACH2 cells were incubated with CPI-637 (20 μM) for 48 h, recruitment of CDK9 and cyclin T1 by BRD4 was analyzed by co-IP **(E)** and the occupancy of HIV-1 Tat, P-TEFb, RNA Pol II CTD phosphorylation at the HIV-1 LTR promoter was determined by ChIP-qPCR assays **(F)**. The quantifications of CDK9 phosphorylation on Thr186, CTD-Ser2 and p24 were performed in **(C, D)** The quantifications of CDK9 and cyclin T1 were performed in **(E)** (*p < 0.05 vs DMSO control group, **p < 0.01 vs DMSO control group).

The BRD4 protein plays a role in silencing HIV-1 gene transcription by competing with the HIV-1 Tat protein for binding to P-TEFb. BRD4 inhibitors drive the release of sequestered P-TEFb by dissociating BDR4 from the HIV-1 promoter and recruiting Tat to stimulate HIV-1 elongation (Bartholomeeusen et al., 2012; Bunch et al., 2019). Therefore, we speculated that CPI-637 may promote viral transcription extension by inhibiting BRD4 protein binding and enhancing the binding of HIV-1 Tat and the P-TEFb complex. As shown in **Figures 4C, D**, CPI-637 clearly increased the phosphorylation of CDK9 on Thr186 and RNA Pol II CTD phosphorylation on Ser2 in a dose- and time-dependent manner. After treatment with CPI-637 for 48 h, recruitment of the P-TEFb subunits CDK9 and cyclin T1 by BRD4 was obviously decreased in ACH2 cells, as shown by co-IP assay (**Figure 4E**), indicating that CPI-637 promoted HIV-1 transcription by a P-TEFb pathway.

The HIV-1 Tat protein is a key transactivator that activates HIV-1 LTR-directed transcription by recruiting P-TEFb to transactivation response (TAR) RNA (Spector et al., 2019). Here, we evaluated the regulatory effect of CPI-637 on expression of the Tat protein. TZM-bl cells stably transfected with a plasmid expressing Tat or an empty vector were tested by WB assay. The Tat-overexpressing efficiency of TZM-bl cells was shown in **Figure 4A**. Integrated luciferase reporter gene expression driven by the HIV-1 LTR in TZM-bl^+Tat^ cells or normal TZM-bl cells was detected with a microplate reader. As shown in **Figure 4B**, LTR-driven activation after treatment with CPI-637 for 48 h was significantly increased up to 67.3-fold in the TZM-bl^+Tat^ cells, whereas Tat increased luciferase activity by only 29.7-fold relative to that in the mock control. Here, CPI-637 increased luciferase activity in normal TZM-bl cells by approximately 3.7-fold compared to that in the mock control group.

Furthermore, the effects of CPI-637 on the HIV-1 Tat, P-TEFb, and phosphorylated RNA Pol II CTD occupancy at the HIV-1 promoter were quantitatively determined in ACH2 cells by ChIP-qPCR assays. As shown in **Figure 4F**, CPI-637 induced a significant decrease (by 1.9-fold compared to the DMSO control group) in the level of BRD4 bound to the HIV-1 promoter, while the levels of CDK9, Tat, and RNA Pol II CTD Ser2 phosphorylation were upregulated by 4.2-fold, 7.2-fold and 6.0-fold, respectively. These results indicate that CPI-637 reactivated HIV-1 expression by dissociating BRD4 from the HIV-1 LTR promoter, permitting Tat to bind P-TEFb and assembling Tat-SEC on the TAR stem-loop structure for RNA Pol II CTD Ser2 phosphorylation.

### CPI-637 Activates HIV-1 Latency *via* a TIP60-Mediated Signaling Pathway

**Figure 5:**
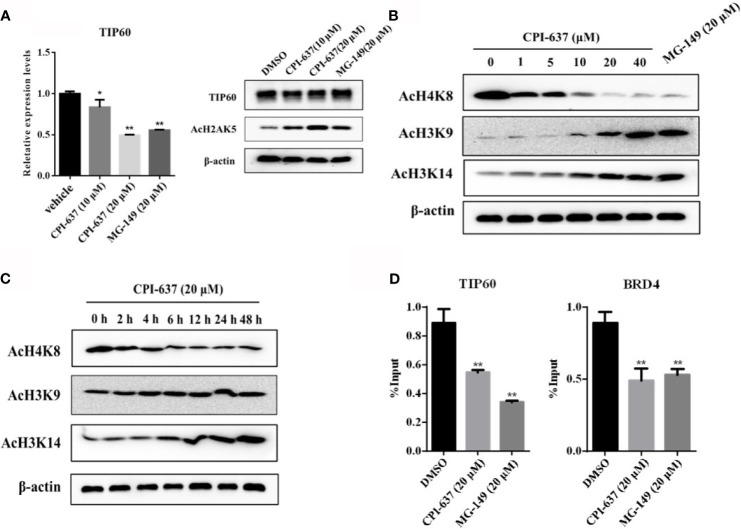
CPI-637 activates HIV-1 latency *via a* TIP60-mediated signaling pathway. The effect of CPI-637 at the indicated concentrations on the expression of TIP60 mRNA and AcH2AK5 level were determined by RT-qPCR and WB respectively **(A)**. ACH2 cells were treated with CPI-637 for 48 h at the indicated concentrations **(B)** or with CPI-637 (20 μM) at the indicated time points **(C)**, and then the levels of AcH4K8, AcH3K9 and AcH3K14 were analyzed by WB. After treatment with CPI-637 (20 μM) for 48 h, the occupancy of BRD4 and TIP60 at the HIV-1 LTR promoter in ACH2 cells was detected by ChIP-qPCR assays **(D)**. (*p < 0.05 vs DMSO control group, **p < 0.01 vs DMSO control group).

Notably, BRD4 is recruited to the HIV-1 LTR by interactions with AcH3 and AcH4. The HIV-1 LTR characteristically has low AcH3 but high AcH4 content, which attracts BRD4 to suppress the interaction of Tat and host SEC. TIP60 was found to promote HIV-1 latency by enhancing the AcH4 level, and the consequential antagonization of TIP60 activates Tat-dependent HIV-1 transcriptional elongation and removes AcH4 and BRD4 from the LTR (Creaven et al., 1999; Li et al., 2018). The reactivation effect of CPI-637, a TIP60 inhibitor, might be attributed to regulation of the expression of TIP60. Here, we first examined the effect of CPI-637 on TIP60 expression in ACH2 cells by RT-qPCR and WB analysis. As shown in **Figure 5A**, treatment with CPI-637 for 48 h significantly inhibited TIP60 mRNA expression but not affected TIP60 protein expression. However, we observed an obviously increase of the level of acetylated H2AK5 in the presence of CPI-637, which is an endogenous marker for TIP60 activity (Achour et al., 2009). This indicates that CPI-637 may reactivate latent HIV-1 by a TIP60-mediated signaling pathway. Our results further confirmed that CPI-637 decreased AcH4K8 level in a dose- and time-dependent manner but clearly increased AcH3K9 and AcH3K14 level (**Figures 5B, C**). Additionally, CPI-637 stimulation induced a significant decrease in TIP60 and BRD4 bound to the HIV-1 promoter, as shown by a ChIP assay (**Figure 5D**). A TIP60 inhibitor, MG-149, was chosen as a positive control. These results indicate that CPI-637-mediated TIP60 downregulation decreased the recruitment of BRD4 and TIP60 to the HIV-1 promoter and reactivated latent HIV-1 by inhibiting the TIP60-AcH4-BRD4 axis.

### CPI-637 Synergistically Reactivates Latent HIV-1 With Conventional Candidate LRAs

**Figure 6:**
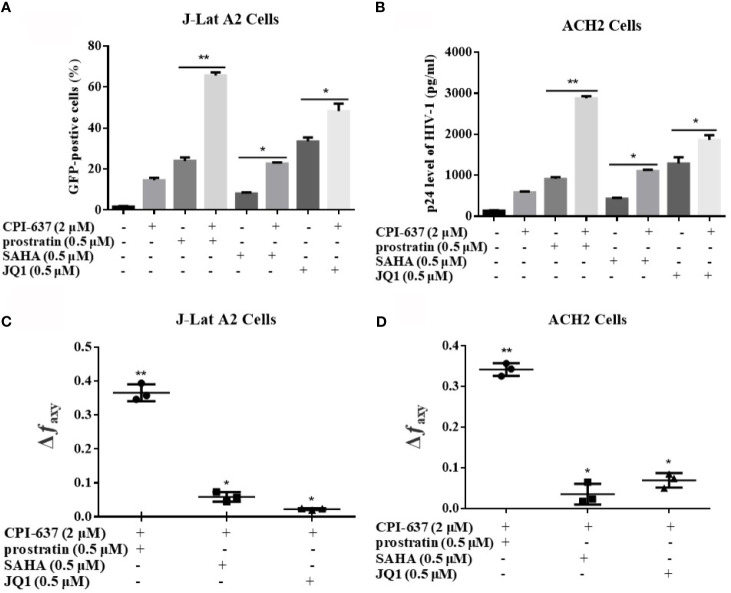
CPI-637 synergistically reactivates latent HIV-1 with conventional candidate LRAs. J-Lat A2 cells **(A)** and ACH2 cells **(B)** were treated with CPI-637 (2 μM), SAHA (0.5 μM), JQ1 (0.5 μM), prostratin (0.5 μM) or their combinations for 48 h. The percentage of GFP-positive cells within the entire population was measured by flow cytometry and HIV p24 antigen expressions were analyzed by ELISA. The Bliss independence model was utilized as a way to calculate the synergy of LRA combinations. Δ*f_axy_* > 0 signifies synergy, whereas Δ*f_axy_* < 0 signifies antagonism. And Δ*f_axy_* = 0 signifies additive effect **(C, D)**. (*p < 0.05 vs combination group, **p < 0.01 vs combination group).

Generally, multiple cell signaling pathways are crucial to the incubation and maintenance of HIV-1 latency. CPI-637, a potential bifunctional candidate LRA, may more efficiently reactivate HIV-1 latency by targeting both the BRD4 and TIP60 proteins. We further assessed the reactivation of latent HIV-1 upon treatment with CPI-637 in combination with some conventional candidate LRAs: the PKC agonist prostratin, HDAC inhibitor SAHA and BET inhibitor JQ1, in different latently infected cell lines. As shown in **Figures 6A, B**, the combination of CPI-637 and prostratin markedly reactivated HIV-1 latency. On the basis of the Bliss independence model described in the Materials and Methods section, the combination of CPI-637 with prostratin was more effective than either alone (Δ*f_axy_* > 0) (**Figures 6C, D**). It is worth mentioning that CPI-637 could not synergistically reactivate latent HIV-1 with SAHA or JQ1 in either J-Lat A2 or ACH2 cells. These results further confirm that CPI-637 might affect the TIP60-AcH4-BRD4 axis. The combination of CPI-637 with prostratin might be an effective way to reverse HIV-1 latency.

## Discussion

The HIV-1 latent reservoir is a major obstacle to the eradication of viral infection. The “shock and kill” strategy aims to reverse HIV-1 latency by regulating host cells and to “kill” reactivated cells through the immune system or anti-HIV-1 drugs (Kim et al., 2018; Abner and Jordan, 2019; Bandera et al., 2019). The first challenge with this strategy is to find ideal LRAs to efficiently reverse latent HIV-1. Unfortunately, none of the LRAs in clinical trials has demonstrated effectiveness in completely curing HIV-1/AIDS due to clinical inefficiency and/or high toxicity (Bashiri et al., 2018; Spivak and Planelles, 2018). There is an urgent need to identify an effective, safe and affordable candidate LRA as a functional HIV-1 cure.

BRD4 is a critical regulator of the establishment of HIV-1 latency (Itzen et al., 2014; Abner et al., 2018). TIP60 is one of the major HATs known to modify H4 and regulate transcription (Zhang et al., 2017; Ghobashi and Kamel, 2018). Recent research shows that downregulation of the nuclear HAT TIP60 expression removed AcH4 and BRD4 from the HIV-1 LTR, resulting in increased SEC loading, Tat transactivation and HIV-1 reactivation. The combination of a TIP60 inhibitor (MG-149) and a BET inhibitor (JQ1) was proven to be a more effective but relatively less toxic strategy to promote HIV-1 reactivation (Li et al., 2018). Therefore, the TIP60-AcH4-BRD4 axis may represent a potential therapeutic target for the development of an ideal LRA. The combined application of different kinds of LRAs or an LRA with dual/multiple mechanisms is a potentially effective strategy for purging latent reservoirs.

CPI-637 is a novel and selective inhibitor of the HATs CBP/EP300 and BRD4 that demonstrates >700-fold selectivity over BRD4 (Taylor et al., 2016). CBP and its close paralog EP300 are two closely related multifunctional transcriptional coactivators (Dutta et al., 2016; Polansky and Schwab, 2018; He et al., 2021). EP300 can maintain the structural stability and HAT activity of TIP60 by facilitating TIP60 autoacetylation, or vice versa (Xiao et al., 2014). Moreover, CBP and TIP60 have distinct and synergistic effects on histone acetylation at H3K9, H3K14, H3K18, H4K5 and H4K12 (Kimura and Horikoshi, 1998). Based on the above findings, we suspect that CPI-637 could be developed as an ideal LRA targeting the TIP60-AcH4-BRD4 axis.

Generally, J-Lat A2, J-Lat 10.6 and ACH2 cells are commonly used as well-established cell models of HIV-1 latently infection *in vitro*. In this study, we found that CPI-637 could potently reactivate latent HIV-1 expression in those three HIV-1 latent cell models with multiple mechanism to maintain HIV-1 latency (**Figure 1**). One of the main contributors to the failure of some LRAs, such as JQ1 and prostratin, in clinical trials may be attributed to their systemic release of proinflammatory cytokines or/and serious toxicity. Moreover, a broad-spectrum HDAC inhibitor, SAHA, induced an increase in the susceptibility of CD4^+^ T cells to HIV-1 (Lucera et al., 2014). Our data showed that CPI-637 exhibited approximately 10-fold lower CC_50_ values than JQ1 in all tested cell lines (**Table 2**) and did not induce either global T cell activation or the release of proinflammatory cytokines (**Figure 3**). It is well known that the expression of inflammatory cytokines in the peripheral blood is clearly increased in HIV-1-infected individuals (Homji et al., 2012; Ahir et al., 2015; Liang et al., 2019), which can cause tissue damage, multiple organ failure and death. We were delighted to find that CPI-637 significantly inhibited the release of both LPS- and prostratin-stimulated proinflammatory and inflammatory cytokines. Thus, CPI-637 might be beneficial for hyperinflammatory conditions in the HIV-1-induced “cytokine storm”. Generally, coreceptors, including CCR5 and CXCR4, help CD4 receptors bind the HIV-1 envelope glycoprotein gp120 and play a vital role in the migration, development and distribution of T cells. Our results showed that CPI-637 drastically downregulated the expression of CCR5 and CXCR4 (**Figure 3**), indicating that it might be advantageous to reduce the susceptibility of CD4^+^ T cells *in vitro* and block viral spread to uninfected bystander CD4^+^ T cells. More extensive animal studies to evaluate the effect of CPI-637 on the infection will be carried out in the future.

Because a single LRA might be insufficient to induce HIV-1 reactivation due to the complexity of the multiple signaling regulatory pathways that establish the HIV-1 latent reservoir (Sadowski and Hashemi, 2019), the use of combinations of LRAs has the potential to be an efficient strategy to activate latent HIV-1 reservoirs and reduce the side effects of using a single LRA. We found that, in agreement with previous studies, the combination of CPI-637 and prostratin was significantly more effective in stimulating HIV-1 expression from latency in J-Lat A2 cells and ACH2 cells than CPI-637 or prostratin alone, indicating that the combination of CPI-637 with other LRAs could effectively purge latent reservoirs with multiple mechanism of HIV-1 latency (**Figure 6**). Prostratin could induce HIV-1 reactivation *via* stimulating IKK-dependent phosphorylation and degradation of IκB, resulting in the rapid nuclear translocation of NF-κB and HIV-1 LTR activation in a NF-κB enhancer-dependent manner. Therefore, CPI-637 or its combination may be reverse latent HIV-1 infection by a NF-κB pathway. Based on these results, CPI-637 shows great potential as an ideal candidate for development as an HIV-1 LRA, and further investigation of the mechanism of CPI-637 as a potential candidate LRA is warranted.

The HIV-1 Tat protein is a key transactivator of viral replication and essential for transcriptional elongation by its binding to the TAR RNA stem loop in the HIV-1 LTR promoter (Asamitsu et al., 2018; Spector et al., 2019). Research has illustrated that JQ1-mediated activation of HIV-1 from latency is attributable to its regulation of the expression of Tat (Itzen et al., 2014). CPI-637, like JQ1, is a BET inhibitor and may reverse HIV-1 latency *via* a Tat-dependent signaling pathway. As expected, our results demonstrated that CPI-637 increased LTR-driven luciferase activity by only 3.7-fold in TZM-bl cells and that LTR-mediated expression by CPI-637 was significantly increased up to 67.3-fold in TZM-bl^+Tat^ cells (**Figure 4**), indicating that Tat plays a critical role in CPI-637-mediated HIV-1 latent reactivation.

P-TEFb, a kinase composed of the catalytic subunit CDK9 and regulatory subunit cyclin T1, plays a critical role in HIV-1 transcriptional elongation by CDK9-mediated phosphorylation of the RNA Pol II CTD on Ser2 (Zaborowska et al., 2016; Fujinaga, 2020). As we described above, the HIV-1 Tat protein has been identified to induce direct interaction with HIV-1 TAR and compete with BRD4 for P-TEFb recruitment to stimulate HIV-1 transcription elongation (Bunch et al., 2019). Therefore, we further tested the effect of CPI-637 on HIV-1 transcription *via* the dissociation of BRD4 from P-TEFb. Compared with J-Lat A2 and 10.6 cell models, ACH2 cells are chronically infected with HIV-1 strain LAI with TAR and can be induced to make infectious virus by a variety of LRAs. Since ACH2 cells after the treatment of LRAs may better mimics the latent reactivation *in vivo*, it is more suitable for the evaluation of the mechanism of LRAs. The results revealed that CPI-637 upregulated the phosphorylation of CDK9 on Thr186 and RNA Pol II CTD on Ser2 in a dose- and time-dependent manner, thereby facilitating HIV-1 transcriptional elongation (**Figures 4C, D**). In addition, the co-IP assay showed that CPI-637 remarkably increased the dissociation of CDK9 and cyclin T1 from BRD4. Additionally, ChIP analysis demonstrated that CPI-637-mediated reactivation downregulated BRD4 but upregulated CDK9, Tat, and RNA Pol II CTD-Ser2P recruitment directly to the HIV-1 LTR (**Figures 4E, F**), indicating that CPI-637 reactivates HIV-1 expression by dissociating BRD4 from the HIV-1 LTR promoter, permitting Tat to bind P-TEFb and assembling Tat-SEC formation on the TAR stem-loop structure for RNA Pol II CTD phosphorylation at Ser2.

Chromatin, an instructive DNA scaffold, is subject to a variety of posttranslational modifications that have a significant impact on histone amino termini in the nuclei of all eukaryotic cells. The chromatin and epigenetic landscape also affects HIV transcription and an active landscape can be maintained by histone methyl transferases (HMT) and HAT. Among the histone modifications, histone acetylation, regulated by HATs and HDACs, play a key role in the organization of chromatin domains and gene expression. The HATs utilize acetyl CoA as cofactor to acetylate the ϵ-amino group of lysine side chains, allowing increased accessibility of transcription factors to DNA and gene transcription activation (Bannister and Kouzarides, 2011). A recent study revealed that HAT TIP60 is a host factor that enforces HIV-1 latency and suppresses HIV-1 reactivation by regulating AcH3 and AcH4 on the viral LTR. Moreover, TIP60-mediated AcH3 downregulation and AcH4 upregulation at the HIV-1 LTR induced BRD4 recruitment to the HIV-1 LTR promoter, where it competes with Tat for P-TEFb, thereby blocking Tat-SEC formation and ultimately inhibiting Tat transactivation (Li et al., 2018). As shown above, CPI-637 is a highly selective dual-target inhibitor of CBP/EP300 and BRD4. In light of the intimate connection between CBP/EP300 and TIP60, we suspected that CPI-637 could reverse HIV-1 latency *via* a second signaling pathway mediated by TIP60. In the present study, we found that CPI-637 is a potent TIP60 inhibitor that downregulated AcH4K8 but upregulated AcH3K9 and AcH3K14 in a dose- and time-dependent manner (**Figures 5A, B**). Importantly, in the ChIP assay, CPI-637-mediated HIV-1 reactivation inhibited BRD4 and TIP60 recruitment to the HIV-1 promoter in accord with the TIP60 inhibitor MG-149 (**Figure 5D**), suggesting that CPI-637 also promotes HIV-1 expression *via* the TIP60-AcH3 and AcH4-BRD4 pathways in the absence or presence of host transcription factors and transcription repressors.

Taken together, our results amply confirm that CPI-637 is an ideal potential bifunctional candidate LRA whose effects are mediated by both BRD4 and TIP60 with high reactivation efficacy and low toxicity. There is a positive synergistic effect between the two signaling pathway of CPI-637 on HIV-1 reactivation. CPI-637-mediated TIP60 downregulation might further stimulate BRD4 dissociation from the HIV-1 LTR promoter *via* AcH3 upregulation and AcH4 downregulation, allowing Tat to more effectively bind P-TEFb and facilitating Tat-SEC formation compared to BRD4 inhibition alone. In addition, the combination of CPI-637 and prostratin more efficiently promoted HIV-1 expression in HIV-1 latency cell models than CPI-637 or prostratin alone, which might be closely related to the complementarity between the PKC signaling pathway and dual mechanisms of CPI-637. CPI-637 opens up new prospects for novel dual-target inhibitors to antagonize HIV-1 latency and deserves further investigation for development as a promising LRA for HIV-1 eradication.

## Data Availability Statement

The raw data supporting the conclusions of this article will be made available by the authors, without undue reservation.

## Author Contributions

LL, TZ, CZ, and JQ contributed to conception and design of thestudy. TZ, CZ, PC, ZW, and YH organized the database. PC, ZW, and YH performed the statistical analysis. TZ wrote the first draftof the manuscript. LL, TZ and JQ wrote sections of the manuscript. All authors contributed to the article and approved the submitted version.

## Funding

This study was supported by the Natural Science Foundation of China (82073896 and 81673481 to LL), Guangdong Basic and Applied Basic Research Foundation (2019A1515010061 and 2021A1515011096 to LL), Natural Science Foundation of Guangdong Province (2018B030312010 to LL), Guangzhou Science and Technology Basic and Applied Basic Research Foundation (202102080108 to LL), and Opening Project of Zhejiang Provincial Preponderant and Characteristic Subject of Key University (Traditional Chinese Pharmacology) at Zhejiang Chinese Medical University (ZYAOXZD2019001 to LL).

## Conflict of Interest

The authors declare that the research was conducted in the absence of any commercial or financial relationships that could be construed as a potential conflict of interest.
